# A sex specific approach of ophthalmic and middle cerebral arteries Doppler in smokers

**DOI:** 10.1038/s41598-021-00503-3

**Published:** 2021-11-05

**Authors:** Maria Marta B. M. Paes, Luísa Macedo Mendes Martins, Angélica L. D. Diniz

**Affiliations:** 1 Department of Ultrasound, Clinical Hospital of the Federal University of Uberlândia, State of Minas Gerais, Brazil; 2 Faculty of Medicine, UNIUBE University, Uberaba, Brazil; 3 Department of Gynecology and Obstetrics, Clinical Hospital of the Federal University of Uberlândia, Av. Pará, SN - Umuarama, Uberlândia - MG, State of Minas Gerais, 38405-320 Brazil

**Keywords:** Medical research, Neurology

## Abstract

Vascular dysfunctions can progress and lead to stroke and cardiovascular disease, especially in smokers. The presence of particular vascular changes according to sex has been described and they can be identified by the Doppler method. This study evaluated Doppler velocimetry parameters of the Ophthalmic Artery (OA) and the Middle Cerebral Artery (MCA) according to sex in smokers regarding a non-smoker group. This cross-sectional observational study included 178 subjects: 93 women and 85 men. Doppler parameters were assessed in OA and MCA. Student’s t-test was used, with p < 0.05. There were no significant differences in OA and MCA Doppler velocimetry data between male non-smokers and smokers. However, female smokers presented several differences compared with non-smokers: lower pulsatility index (PI) and higher peak ratio in OA, and higher PI and resistance index and lower end diastolic velocity in MCA. There were different brain vascular waveforms in the group of female smokers compared with non-smokers. Cigarette smoking also led to opposite arterial patterns in OA and MCA in the female group, with signs of falling impedance in OA and increased impedance in MCA. An individualized approach regarding arterial changes according to sex is desirable.

## Introduction

Vascular dysfunctions are a burden on global public health, as they can cause cardiovascular diseases (CVD), stroke, and dementia, which lead to high rates of morbidity and mortality^1,2^. There are non-modifiable risk factors for developing these vascular diseases, including age, sex, low birth weight, race/ethnicity, and genetic predisposition. However, there are well-documented modifiable risk factors, among which cigarette smoking and hypertension are worth highlighting^2^.

Cigarette consumption confers a 25% increased risk of coronary heart disease in women compared with men as well higher risk of haemorrhagic stroke, independently of differences among other risk factors^3,4^. In a recent meta-analysis including 3,980,359 individuals, the authors described that smoking was an independent risk factor for stroke in both sexes, but when a regional analysis was made there was evidence of a higher harmful effect of smoking in women than in men in the western population^4^. Although women and men share most of the classic risk factors, there are factors that cause different and stronger impacts on women, such as smoking, diabetes, high triglycerides and low levels of HDL-cholesterol^5^. Based on this situation, there is a need to study the cerebral vascular pattern with a focus on the findings according to sex.

Both CVD and stroke are dysfunctional vascular diseases whose first stages can be subtle or even subclinical and asymptomatic^6^. The vascular tree is composed of distinct segments where each blood vessel has a different calibre, and vascular walls have their own regional characteristics and blood flow velocity; these features mean that they have a particular waveform, or individual signature, when examined with Doppler ultrasound. This method can evaluate arterial waveform and quantify its velocity and impedance changes in real time^6^. The middle cerebral artery (MCA) is an intracranial vessel whose changes in the blood flow waveform are correlated with diseases of small vessels^7^. The ophthalmic artery (OA) has intra- and extra-cranial connections that can indicate pre-clinical stages of disease before it becomes clinically apparent^6^. We are unaware of research that has used Doppler ultrasound for both the OA and MCA in the evaluation of cerebral haemodynamic changes in smokers according to sex. Thus, this study contributes to a better understanding of cerebral vascular physiology in this population, as well as the possibility of individual assistance in both men and women.

The aim of this study was to compare Doppler parameters of the OA and the MCA between smoking and non-smoking subjects, according to sex.

## Methods

### Study design

This was a cross-sectional, observational study, conducted at the Clinical Hospital of Uberlândia, Brasil, and was approved by the local ethics committee, Plataforma Brasil—Universidade Federal de Uberlândia, under number 22088713.3.0000.5152. All procedures followed the Declaration of Helsinki, and all patients provided informed consent to participate in the study.

### Study population selection

The study sample comprised 180 volunteers, among whom 95 were women (56 non-smokers and 39 smokers) and 85 were men (51 non-smokers and 34 smokers). The smoker group (SG) was designed with 71 volunteers and the control group (CG) with 107 volunteers. The inclusion of the volunteers was consecutive and for convenience, and the inclusion criteria were: age between 18 and 50 years, presenting no known or reported diseases during anamnesis and in medical records, that is, diabetes, hypertension, dyslipidemia, migraine, history of stroke and vasculopathies, normal body mass index (20–25 kg/m^2^), and women in menacme. Among smokers, those who smoked at least five filtered cigarettes a day for at least 2 years were included. The exclusion criteria were: use of systemic or neurological medications, including antihypertensive, anticoagulant, antiarrhythmic, and anticonvulsant drugs; volunteers that consumed regularly illicit drugs and/or alcohol; and consumption of illicit drugs and/or alcohol up to 72 h before the Doppler exam. In the SG, the number of cigarettes consumed each day and the number of years of smoking were recorded. To assess the degree of nicotine dependence, the Fagerström Test for Nicotine Dependence (FTND) was applied; it comprises a questionnaire with six questions with scores from 0 to 2 and from 0 to 3, with higher scores indicating greater dependence on nicotine. The degree of nicotine addiction was divided into two groups: low to medium when the score was ≤ 5 and high to very high when the score was ≥ 6.

### Doppler tests and data sampling

The OA and the MCA were assessed on the right and left sides of the body and the mean of the two measurements was considered for analysis. A Doppler scan of the OA and the MCA was performed at a single time in the CG during the morning. In the SG, a Doppler scan of the OA and the MCA was performed during the morning, at least 2 h after smoking; this measure was considered baseline (BL)^8^. The length of time since the last cigarette prior to the Doppler exam was recorded. All participants also were free from consumption of coffee, tea or energy drinks for at least 12 h before the Doppler exam and they did not use contact lenses.

The Sonara/tek™ Transcranial Doppler Module was used for MCA Doppler. The transductor was positioned in the transtemporal acoustic window between the angle of the eye and ear above zygomatic and the artery was detected 4–6 cm below the surface where its flow presents relatively constant velocities^9^. Doppler exam in of the OA was performed using a Medison Sonoace 8000SE. The OA was always assessed medial to the optic nerve, using a 7.5 MHz linear transducer, keeping the angle close to 0° and, imperatively, below 20°, a 50 Hz filter, a 5 kHz frequency of pulse repetition, a 2 mm sample volume, and a mechanical index < 1.0^10^. One experienced physician performed all the exams and the following parameters in the OA and the MCA were analysed: resistance index (RI), pulsatility index (PI), peak of velocity systolic (PVS), end diastolic velocity (EDV), and mean velocity (MV). In the OA, the second systolic peak (P2) and the PVS/P2 ratio (PR) were also measured^10^. All Doppler parameters except PR were automatically determined by both pieces of equipment.

### Other clinical variables

Blood pressure, including systolic blood pressure (SBP) and diastolic blood pressure (DBP), was measured on the left upper limb; this occurred automatically in all subjects. Simultaneously to that measurement, Doppler exams and heart rate were automatically determined by the Sonara/tek™ equipment.

### Statistics

The sample size calculation was performed using G*Power software, version 3.1, considering Student’s t-test, a large effect size of 0.8, an alpha level of 0.05, and 90% power. A total of 34 volunteers were calculated for each group. Statistical analysis was performed using SPSS Statistics for Windows version 21.0 (IBM Corp., Armonk, NY, USA) and Jamovi (Version 2.0.0) Computer Software (The jamovi project (2021, Sydney, Australia). Data are reported as number and percentage or mean ± standard deviation (SD). The Shapiro–Wilk test was used to assess whether the quantitative variables were normally distributed. Student’s t-test for independent samples was used to compare the means of the SG and CG. For all analyses, p < 0.05 was considered to indicate a statistically significant difference and CI ≥ 95%. The effect size was estimated by the Cohen d test, adopting non-significant if test between 0.00–0.19, small, moderate and high effect if between 0.20–0.49; 0.50–0.79 and 0.80–1.30, respectively. To correct the age and oral contraceptives influence on Doppler indexes, multiple analysis of covariance was applied to statistically significant variables.

## Results

### Clinical data matching

The age for men was 31 ± 9 years in the CG and 32 ± 8 years and in the SG. The age for women was 37 ± 6 years in the CG and 38 ± 9 years in the SG; there were no signi¦cant differences between the sexes (p > 0.05). There was no significant difference in the number of cigarettes smoked per day between men (18 ± 5) and women (15 ± 12) or the previous years of smoking, represented for 16 ± 9 years for men and 20 ± 10 years for women. The cigarette withdrawal time, which was controlled in this study, was 192 ± 108 min for men and 217 ± 129 for women; it was not different between the sexes. Regarding the degree of nicotine addiction based on the FTND, there were 38 volunteers with a score ≤ 5 (23 women and 15 men) and 33 with a score ≥ 6 (14 women and 19 men). Among the female SG, 9 were using oral contraceptives, compared with 12 in the female CG. For men, there were no signi¦cant differences between the SG and the CG for SBP (120 ± 10 and 118 ± 9, p = 0.13), DBP (81 ± 9 and 77 ± 10, p = 0.12), and heart rate (60 ± 10 in both groups). Similarly, for women there was no signifcant difference between the SG and the CG for SBP (110 ± 10 in both groups), DBP (74 ± 9 and 80 ± 9, p = 0.08), and heart rate (66 ± 9 and 63 ± 8, p = 0.15).

The means and standard deviations of ages, patterns of cigarettes consumption, use of oral contraceptives and arterial pressure was compared between men and women smokers and non-smokers groups and are described in Table 1.

**Table 1:** Comparison between clinical variables and patterns of cigarette consumption observed between men and women smokers and non-smokers groups.

Clinical variable	Men (CG)	Men (SG)	Women (CG)	Women (SG)	p value
Age (years) ± sd	31 ± 9	32 ± 8	37 ± 6	38 ± 9	ns
Cigarettes smoked per day ± sd		18 ± 5)		15 ± 12	ns
Previous years of smoking ± sd		16 ± 9		20 ± 10	ns
Cigarette withdrawal time ± sd		192 ± 108		217 ± 129	ns
FTND ≤ 5		15		23	0.03*
FTND ≥ 6		19		14	
Oral contraceptives			12	9	ns
SBP (mmHg) ± sd	120 ± 10	118 ± 09	110 ± 10	110 ± 10	ns
DBP (mmHg) ± sd	81 ± 09	77 ± 10	74 ± 09	80 ± 9	ns
HR (bpm) ± sd	60 ± 10	60 ± 10	66 ± 9	63 ± 8	ns

*CG* control group, *SG* smokers group, *SBP* systolic blood pressure, *DBP* diastolic blood pressure, *HR* heart rate, *FTND* Fagerström Test for Nicotine Dependence, *ns* not significant.

### Doppler study

**Figure 1:**
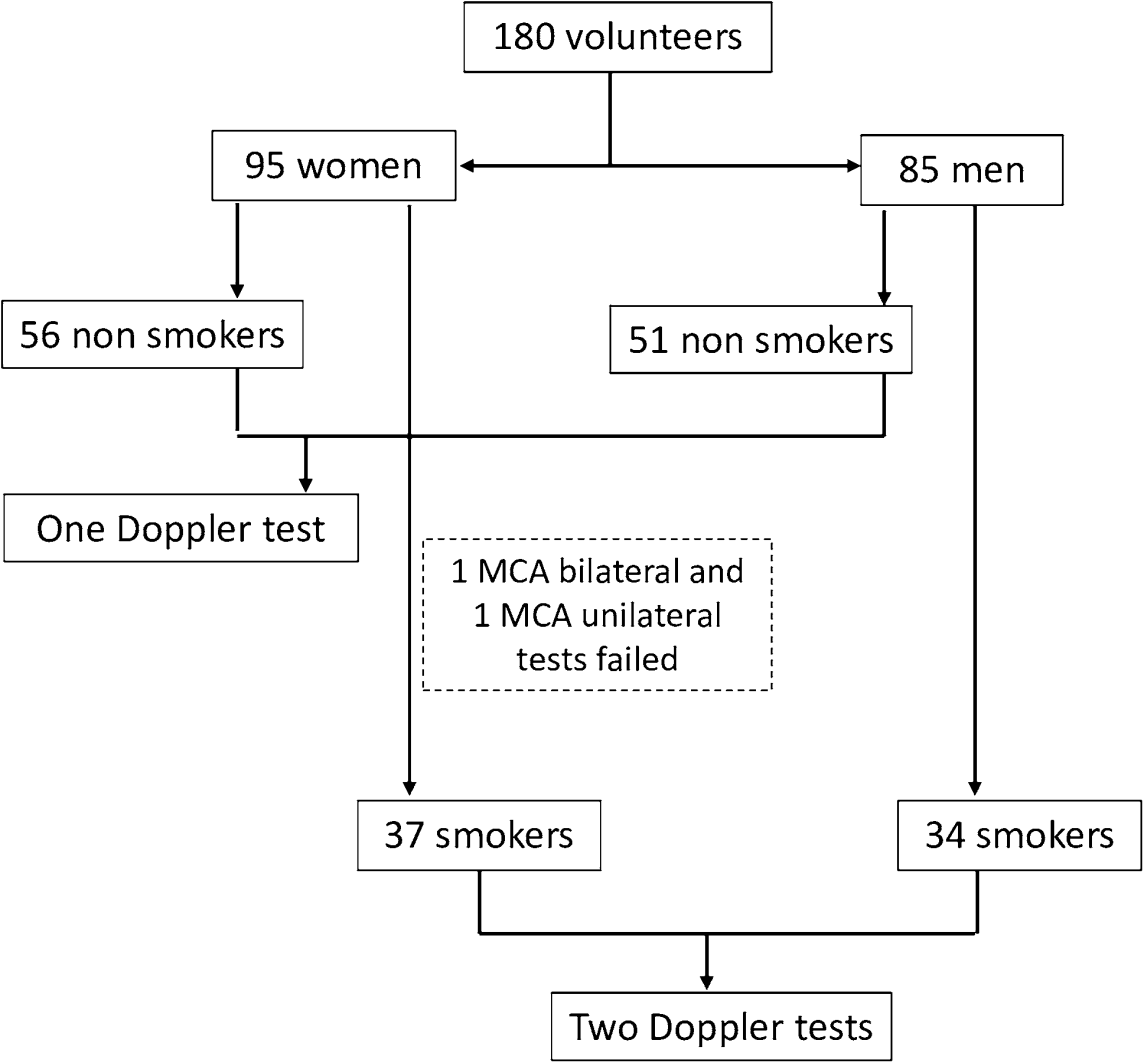
Flowchart showing the inclusion of smokers and controls in this study.

Two female smokers were excluded due to the impossibility of performing the MCA Doppler exam (Fig. 1). Hence, there were a total of 712 arteries scanned in this study (two OA and two MCA per subject). There were no significant differences in men when comparing the OA and the MCA Doppler indexes between the CG and the SG (Table 2).

**Table 2:** Comparison between means and standard deviation of Doppler indexes in the ophthalmic and middle cerebral arteries of smoking and non-smoking men.

	Doppler parameters	Mean	Sd	CI	95%	p-value
				Lower bound	Upper bound	
SG	RI OA	0,76	0,08	0,74	0.77	ns
CG	RI OA	0,77	0,06	0.76	0.78	
SG	PI OA	1,82	0,56	1,69	1,94	ns
CG	PI OA	1,94	0,48	1,83	2,03	
SG	PVS OA cm/s	28,96	6,69	27,43	30,79	ns
CG	PVS OA cm/s	30,68	7,02	29,33	32.03	
SG	P2 OA cm/s	18,94	5,60	17,69	20,42	ns
CG	P2 OA cm/s	19,08	5,59	17,98	20.17	
SG	PR OA	0,66	0,15	0.62	0,69	ns
CG	PR AO	0,62	0,13	0.59	0.64	
SG	EDV OA cm/s	6,72	2,63	6,19	7,30	ns
CG	EDV OA cm/s	7,03	2,01	6,58	7,47	
SG	MV OA cm/s	12,96	3,97	12,15	13,93	ns
CG	MV AO cm/s	12,69	3,43	11,96	13,40	
SG	RI MCA	0,57	0,07	0,55	0,58	ns
CG	RI MCA	0,57	0,06	0,55	0.58	
SG	PI MCA	0,91	0,19	0,86	0,95	ns
CG	PI MCA	0,90	0,17	0,86	0,93	
SG	PVS MCA cm/s	77,28	14,90	73,33	81,42	ns
CG	PVS MCA cm/s	77,99	17,63	74,73	81,23	
SG	EDV MCA cm/s	32,40	7,67	30,27	34,46	ns
CG	EDV MCA cm/s	32,70	9,16	31,01	34,37	
SG	MV MCA cm/s	49,18	9,47	46,45	51,85	ns
CG	MS MCA cm/s	49,87	12,01	47,69	51,85	

*OA* Ophthalmic Artery, *MCA* Middle Cerebral Artery, *MV* mean velocity, *RI* Resistance Index, *PI* Pulsatility Index, *PVS* Peak of Velocity Systolic, *P2* second peak of velocity, *PR* peak ratio, *EDV* End Diastolic Velocity, *SG* study group, *CG* control group, *cm/s* centimeter/second, *sd* standard deviation, *ns* non significant.

However, in women there were significant differences in several of the Doppler indexes between the CG and the SG. In the SG, there was a drop in arterial impedance in the OA, detected by lower PI and higher P2 and PR values. To determine the influence of cigarette use, even with the confounding factors like age and use of oral contraceptives, a multivariate analysis of covariance was performed for the variables that showed statistical significance and the corrected p was discriminated against. Effect size was also estimated by Cohen's, and the results are described in Table 3.

**Table 3:** Comparison between means and standard deviation of Doppler indexes in the ophthalmic and middle cerebral arteries of smoking and non-smoking women.

	Doppler Parameters	Mean	sd	CI	95%	p- value	p- value after MANCOVA	Cohen's d
				Lower Bound	Upper Bound			
SG	RI OA	0,76	0,06	0,75	0,77	ns		
CG	RI OA	0,77	0,05	0,76	0,77			
SG	PI OA	1,62	0,36	1,52	1,71	0.008	smoke: < 0.001	0.547
CG	PI OA	1,84	0,40	1,78	1,92		age: < 0.001 ocp: 0.74	
SG	PVS OA cm/s	26,59	6,25	25,05	28,12	ns		
CG	PVS OA cm/s	28,83	5,80	27,83	29,84			
SG	P2 AO cm/s	20,47	5,23	19,03	21,70	ns		
CG	P2OA cm/s	19,61	6,29	18,26	20,02			
SG	PR OA	0,77	0,15	0,73	0,80	0.001	smoke: < 0,001	0.777
CG	PR OA	0,66	0,13	0,63	0,68		age: < 0.001 ocp: 0.227	
SG	EDV AO cm/s	6,32	2,00	5,81	6,77	ns		
CG	EDV AO cm/s	6,59	1,96	6,32	6,94			
SG	MV OA cm/s	12,87	3,22	11,95	13,58	ns		
CG	MV AO cm/s	12,34	3,54	11,96	13,02			
SG	RI MCA	0,60	0,06	0,58	0,61	0,001	smoke: 0.003	− 0.429
CG	RI MCA	0,57	0,06	0,56	0,58		age:0.740 ocp: 0.217	
SG	PI MCA	0,94	0,18	0,90	0,97	0,001	smoke: 0.006	− 0.397
CG	PI MCA	0,87	0,13	0,85	0,90		age:0.666 ocp: 0.107	
SG	PVS MCA cm/s	78,62	19,19	74,56	82,68	ns		
CG	PVS MCA cm/s	79,03	15,43	77,43	82,77			
SG	EDV MCA cm/s	31,08	10,03	28,90	33,24	0,039	smoke: 0.041	0.291
CG	EDV MCA cm/s	34,64	8,91	32,35	35,21		age: 0.336 ocp:0.076	
SG	MV MCA cm/s	51,04	13,50	48,15	53,91	ns		
CG	MV MCA cm/s	52,90	11,74	50,91	54,70			

*OA* Ophthalmic Artery, *MCA* Middle Cerebral Artery, *MV* mean velocity, *RI* Resistance Index, *PI* Pulsatility Index, *PVS* Peak of Velocity Systolic, *P2* second peak of velocity, *PR* peak ratio, *EDV* End Diastolic Velocity, *SG* study group, *CG* control group, *cm/s* centimeter/second, *ocp* oral contraceptive, *sd* standard deviation, *ns* non significant.

**Figure 2:**
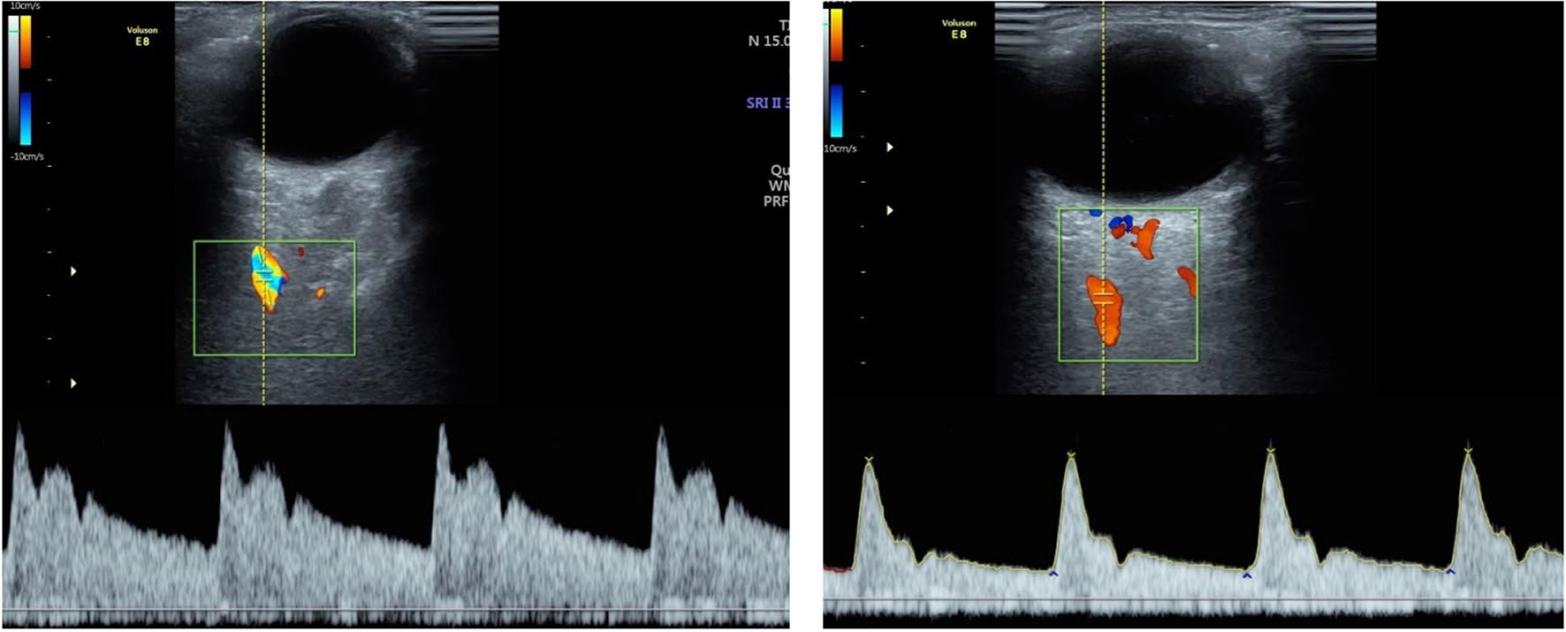
Ophthalmic artery Doppler demonstrating different wave morphology with elevation of the second peak systolic velocity P2 and peak ratio (on the left) compared to Doppler waveform with low P2 and peak ratio in non-smokers group (on the right).

The elevation of PI, P2, and PR modified the wave morphology, with an increase in the hump in relation to the velocity waveform patterns found in the CG (Fig. 2). There were no differences in the other OA Doppler indexes (IR, PVS, and EDV) between the SG and the CG. There was significantly higher impedance in the MCA in the female SG compared with the CG, represented by higher RI and PI and lower EDV. Other velocities (PVS, EDV, and VM) were not different between the two groups. Therefore, there were antagonistic response patterns of the OA and the MCA in the female chronic smokers. Although age had an influence on OA values, cigarette use is an independent modifying factor in wave morphology, since p remains significant even with correction of the covariate age and use of oral contraceptives.

## Discussion

### Vascular waverform changes according to sex

The main findings of the current study were the identification of different vascular waveforms in the female and male SG when properly compared with their same sex CG. A drop in impedance was detected in the territory of the OA and an increase in impedance in the MCA in the female SG compared with the CG. However, there were no signi¦cant differences between the male SG and CG in the vascular waveforms. These findings characterise distinct patterns according to sex. Sex is one among many risk factor for vascular diseases^2^ that had historically been undervalued until recently. Individual factors involving sex and the perception that each one can require different management diagnosis and therapeutics occurred only in the early 2000s, when the ‘one size fits all’ approach started to become invalid^11^. Sex-specific differences in heart diseases can occur because women have sex-specific risk factors related to pregnancy disorders, gestational diabetes, hypertension, preeclampsia, and endocrine changes^5,11^. From the perspective of vascular diseases, sex differences have profound implications for effective stroke prevention and treatment^12^. This is based on the multifactorial nature of anatomic, genetic, and sex hormonal factors^5^. Pre-menopausal women seem less vulnerable to stroke than men of similar ages, but after menopause, there is an increase in stroke among women^12^. When associated with sex, smoking can modify this epidemiology of vascular diseases^1,2^. Prolonged smoking, besides being one of the most relevant modifiable risk factors^2^, also appears to be the most specific risk factors associated with sex in CVD, causing more harm in women than in men^11^ and greater risk of haemorrhagic stroke in women than men, independently of differences in other risk factors^4^.

We did not identify studies in the literature that analysed the Doppler waveforms of both the OA and the MCA by comparing groups of smokers and non-smokers according to sex. Hence, there is not a similar study to which we can compare our data. There is a published study that compared the Doppler parameters of the OA in chronic smokers in relation to a control group of non-smokers, but the researchers used mixed-sex groups12. Their smoker group comprised 49 (27 male, 22 female) cigarette smokers (≥ 2 years and ≥ 10 cigarettes a day), and their control group was 40 healthy non-smokers. The findings were higher RI and lower EDV in the OA in chronic smokers compared with the control group, which is not in accordance with our study, where we identi¦ed lower RI and no signi¦cant difference in the EDV in the group of women, and no differences in men. With regard to the MCA Doppler parameters, researchers have assessed the reactivity of the artery by comparing the findings before and after smoking in the same group, an approach that limits the comparative analysis with our data^13–15^.

(Although the mechanisms that cause vascular diseases have not yet been fully elucidated) Vascular diseases can start linking inflammatory signals initiated and amplified in the microvasculature with the development and progression of atheroma in large vessels, with the inflammation and damage to endothelium^6^, the decrease in the release of nitric oxide in response to shear stress and stretch of vasculature^13^, activation of vascular remodelling^14^, and an increase in arterial wall stiffness^15^ have been implicated. These events cause pathological adaptive changes in the microcirculation and are primordial lesions that increase risks for future macrovascular events and consequent damage to final organs such as the heart and brain^6^. Changes in the arterial waveform of smokers can indicate a sign of the preclinical phase of vascular disease before atherosclerosis, which manifests itself in more advanced stages of vascular impairment^6^. Our findings suggest that in the case of cigarette consumption, women may have worse adaptation in the microcirculation than men. There are several other causes that can justify these findings, such as reproductive endocrine disorders, including pregnancy-induced hypertension, preeclampsia, gestational diabetes mellitus, preterm birth; Also, these differences are mainly caused by innate genes and environmental influences^5^.

In relation to PR, which represents an index of the morphology waveform, the exact mechanism of its modification in the female SG is still unclear. Its increase has been correlated to hyperperfusion^16^ or to waveform change caused by the sum of the incident wave travelling towards the periphery and the early return of the reflected wave returning from the periphery reflecting on the systole and raising P2^15^. This increased reflection is caused by arterial stiffness and increased local vascular tone, which cause repetitive reflections of the waves along the systemic arterial tree between the heart and the arteries studied^17^. Therefore, the higher PR may reflect local arterial preclinical disease and its possibility for future progression to atherosclerosis^18^, early changes in microcirculatory compliance causing downstream hyperperfusion, or both. In female smokers, PR is increased, which may suggest arterial stiffness and/or distal hyperperfusion in women.

Of note, only the female SG presented elevated RI and PI in the MCA in relation to the CG. The increase in PI in the MCA has been correlated with impaired cerebral perfusion due to small vessel disease^19^ and worse cognitive performance related to microangiopathy and inflammation^20^. In addition, PI in the MCA assessed by transcranial Doppler is well correlated with a variety of manifestations observed by magnetic resonance imaging, such as periventricular hyperintensity, deep white matter hyperintensity, lacunar disease, and dot hyperintensity; this factor strengthens the hypothesis that this index reflects the degree of resistance downstream in the intracranial circulation and that its values are high in small vessel diseases^7^.

Both vasospasm in basal cerebral arteries and dilation of peripheral vessels or even a combined effect of both have been postulated as actions of cigarette components^21^. Only female smokers showed a statistically significant increase in RI and PI of the MCA. Besides, an increase in EDV is regarded as an indirect sign of dilation of smaller vessels, associated with a decrease in peripheral resistance^22^; thus, its reduction in the female SG is yet another sign of reduction in cerebral peripheral perfusion.

Regarding both arteries studied, the OA showed an opposite change in waveform to the MCA. The reasons for these ¦ndings may be due to the difference in calibre between these vessels, diferente mechanisms of self-regulation, or complex interactions between these vessels24. Although the AO has been used to evaluate cerebral circulation25, we found a different waveform pattern in relation to the MCA because the OA may attempt to compensate for the cerebral blood §ow through increased perfusion or vasodilation. The waveform changes in the OA may re§ect a compensatory mechanism known as ‘inverse steal’ of this vessel, characterised by increased blood §ow in this vessel in an attempt to increase cerebral blood §ow when it is reduced due to vasoconstriction of local arterioles26. Thus, the altered AO and MCA waveforms in female smokers may be due in part to vasospasm of conductive cerebral vessels as well as vasoconstriction of small cerebral vessels.

### MCA and OA interactions

A drop in impedance was detected in the territory of the OA and an increase in impedance in the MCA in the female SG compared with the CG. However, there were no significant differences between the male SG and CG in the vascular waveforms. These findings characterise distinct patterns according to sex.

We did not identify studies in the literature that analysed the Doppler waveforms of both the OA and the MCA by comparing groups of smokers and non-smokers according to sex. Hence, there is not a similar study to which we can compare our data. There is a published study that compared the Doppler parameters of the OA in chronic smokers in relation to a control group of non-smokers, but the researchers used mixed-sex groups^23^. Their smoker group comprised 49 (27 male, 22 female) cigarette smokers (≥ 2 years and ≥ 10 cigarettes a day), and their control group was 40 healthy non-smokers. The findings were higher RI and lower EDV in the OA ​​in chronic smokers compared with the control group, which is not in accordance with our study, where we identified lower RI and no significant difference in the EDV in the group of women, and no differences in men. With regard to the MCA Doppler parameters, researchers have assessed the reactivity of the artery by comparing the findings before and after smoking in the same group, an approach that limits the comparative analysis with our data^22,24^.

Regarding both arteries studied, the OA showed an opposite change in waveform to the MCA. The reasons for these findings may be due to the difference in calibre between these vessels or different mechanisms of self-regulation^25^. Although the OA has been used to evaluate cerebral circulation^26^, we found a different waveform pattern in relation to the MCA because the OA may attempt to compensate for the cerebral blood flow through increased perfusion or vasodilation. The waveform changes in the OA may reflect a compensatory mechanism known as ‘inverse steal’ of this vessel, characterised by increased blood flow in this vessel in an attempt to increase cerebral blood flow when it is reduced due to vasoconstriction of local arterioles^27^. Thus, the altered OA and MCA waveforms in female smokers may be due in part to vasospasm of conductive cerebral vessels as well as vasoconstriction of small cerebral vessels or mainly by compensatory interactions between these two vessels.

### Limitations

The main limitation of this study, as in all studies using Doppler, is the impossibility to evaluate arterial diameter, a factor that limits the ability to clarify the exact mechanism that causes changes in the waveform. Thus, it is not possible to determine whether the biggest changes occur in the conductive vessel by vasospasm or in the downstream microcirculation, or whether there is a combined effect of both. Another limitation was the failure to measure intraocular pressure (IOP) in subjects undergoing the Doppler study. However, it has been described in the literature that acute elevation in IOP has no effect upon ophthalmic artery Doppler indexes and the haemodynamics are unrelated to acute fluctuations of the IOP over a wide range, suggesting that ocular hypertension itself cannot induce vascular dysfunction in this artery^28^.

## Conclusion

In the current study, it was possible to identify different brain vascular waveforms in the female SG compared with the CG, but the same did not occur in the male SG compared with the CG. Thus, women might present worse adaptation to cigarette consumption and more signs of vascular dysfunction. Regarding arteries change, cigarette smoking led to opposite waveform in the OA and the MCA in the female SG, with signs of reduced impedance in the OA and increased impedance in the MCA. These findings allow us to infer that these arteries have complex interactions with each other and thus, different reactivity and adaptations to cigarette use. Therefore, there is a clear need for an individualised approach regarding arterial changes according to sex.
